# A simple method to make, trap and deform a vesicle in a gel

**DOI:** 10.1038/s41598-023-31996-9

**Published:** 2023-04-02

**Authors:** Pierre Tapie, Alexis M. Prevost, Lorraine Montel, Léa-Laetitia Pontani, Elie Wandersman

**Affiliations:** Sorbonne Université, CNRS, Institut de Biologie Paris-Seine (IBPS), Laboratoire Jean Perrin (LJP), 4 place Jussieu, 75005 Paris, France

**Keywords:** Biophysics, Membrane biophysics, Membrane structure and assembly, Permeation and transport, Biomaterials, Biomimetics, Biomaterials, Soft materials, Techniques and instrumentation

## Abstract

We present a simple method to produce giant lipid pseudo-vesicles (vesicles with an oily cap on the top), trapped in an agarose gel. The method can be implemented using only a regular micropipette and relies on the formation of a water/oil/water double droplet in liquid agarose. We characterize the produced vesicle with fluorescence imaging and establish the presence and integrity of the lipid bilayer by the successful insertion of $\alpha$-Hemolysin transmembrane proteins. Finally, we show that the vesicle can be easily mechanically deformed, non-intrusively, by indenting the surface of the gel.

## Introduction

Vesicles have been widely used to mimic cellular compartmentalization and reproduce *in vitro* specific biological functions with bottom-up approaches^1,2^. In many recent studies, the focus was put on the encapsulation of complex biological reactions inside the vesicles, in order to express proteins^3^ or genes^4^, or to reconstitute and study the protein filaments of the cytoskeleton, such as the actin cortex^5^ or microtubule asters^6^. Many other works aim to mimic the cell membrane properties, as for instance membrane fusion^7–9^, or cell communication, via the insertion of transmembrane proteins in liposomes. In particular, mechanosensitive channels^10,11^ have been inserted in the membrane of liposomes, allowing in turn to measure their conductance under mechanical stress.

The production of vesicles usually relies on either hydration methods or inverted emulsion templates. Hydration methods rely on the swelling of dried lipid films in an aqueous buffer^12,13^. They are relatively simple to operate but yield polydisperse vesicle sizes and a relatively non homogeneous encapsulation efficiency^14–16^. The production rate of unilamellar vesicles can be improved by using electric fields (electroformation methods^17^) but fragile proteins can be damaged by the applied fields^18,19^and the technique is also limited to buffers with low ionic concentrations. Inverted emulsion templates, on the other hand, are based on the forced passage of a water-in-oil emulsion droplets through a water/oil interface^5,20^. This method can be developed in microfluidic chips^21–24^, yielding monodisperse vesicle sizes, that are limited by the microfluidic channel dimensions.

In both methods (hydration or emulsions), the resulting vesicles are dispersed in the outer medium, which requires additional steps to handle or transfer them in different environments (micropipettes^25^, optical trapping^26^, etc). These extra steps, relying on specific technical skills, make it difficult to replicate many experiments and collect large statistics on the systems properties. In particular, in order to study mechanotransduction processes, it is required to mechanically stimulate the vesicles. In practice, this has typically been achieved with local membrane deformations (pipette suction^27^, fluid flows^28,29^, AFM^30,31^). However, these methods do not reproduce faithfully the nature of mechanical perturbations in tissues. In addition, they overlook the mechanical coupling between cells and their biological visco-elastic environment.

We propose here a new technique, that provides a versatile platform for the straightforward production of biomimetic pseudo-vesicles (a vesicle with an oily cap on top), but also allows for their trapping and non-intrusive mechanical excitation.

**Figure 1:**
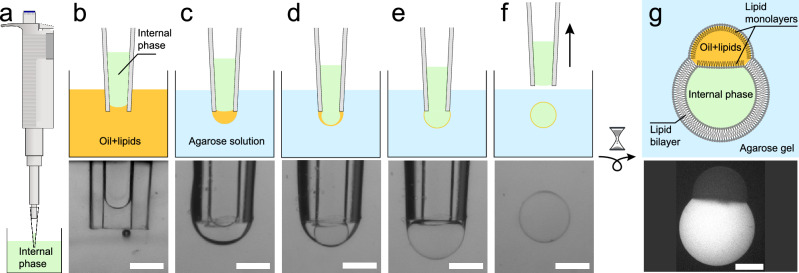
(**a**–**f**) (upper row) Sketch of the double emulsion production method. Green/orange/blue colors stand for internal/oil/external phases, respectively. (bottom row) Bright field images. Scale bars = 500 $\mu$m. The produced double emulsion droplet in(**f**) sediments in the liquid agarose and eventually gets trapped as the agarose gels. Simultaneously, the surrounding oil shell creams at the top of the droplet and a lipid bilayer is zipped on the lower part. A few minutes later, the pseudo-vesicle has been formed and is trapped in the gel, as sketched in (**g**). Bottom panel: Fluorescence macroscope image of a pseudo-vesicle loaded with carboxyfluorescein trapped in an agarose gel. Scale bar = 200 $\mu$m.

## Pseudo-vesicle formation

The vesicles are produced from a water in lipid-containing oil droplet configuration, that is formed in a liquid, warm, agarose solution (details on the chemicals can be found in the “Methods” section). We first draw a small volume (about 500 nL) of the internal aqueous phase (Fig. 1a), using a 2.5 $\mu$L micropipette (Eppendorf). Second, we move the pipette into the oil/lipids container and suck about 50 nL of it (Fig. 1b), by turning the adjustment wheel of the micropipette while the tip is immersed in the oil. The pipette tip thus contains an oil/water sandwich. Last, we move the pipette into the warm agarose solution (temperature $T\approx 38\,^{\circ }C$, see details in the “Methods” section) where we expel the oil/water sandwich by turning backward the adjustment wheel of the micropipette. During this expulsion phase, the oil phase first grows into a droplet in the liquid agarose (Fig. 1c), followed by the aqueous phase that grows inside of it (Fig. 1d,e). Finally, we move up the pipette rapidly in the air, which detaches the double droplet from the tip due to viscous friction from the agarose solution (Fig. 1f).

Immediately after its formation, the inner water droplet is thus surrounded by an oil layer containing phospholipids. These lipids therefore redistribute at both oil water interfaces, with their hydrophilic heads turned towards the inner water phase on one side and towards the agarose solution on the other side. While the agarose solution cools down, the double droplet slowly sediments in the agarose solution and eventually gets trapped in the formed gel. During this cooling process, the oil layer of the double droplet creams under gravity to form an oil cap, while the lipids stabilizing the former oil/water interfaces zip up a lipid bilayer on the lower part of the droplet (Fig. 1g). The double droplet becomes a pseudo-vesicle. This simple micro-pipette method yields pseudo-vesicles with a diameter of 601 $\pm 58 \mu$m (N = 98). Smaller sizes can be obtained using smaller tips and a micro-injector (see “Methods” section). The production rate of the method is around 5 pseudo-vesicles per minute and the rate of success is overall about 30%. Once trapped in the gel, the pseudo-vesicle is stable for several hours and can be kept overnight if the gel-containing cuvette is sealed to prevent evaporation. Moreover, being trapped in the gel, pseudo-vesicles can easily be observed with a microscope, as in^32^.

## Membrane characterisation and functionalization

### Fluorescence imaging

**Figure 2:**
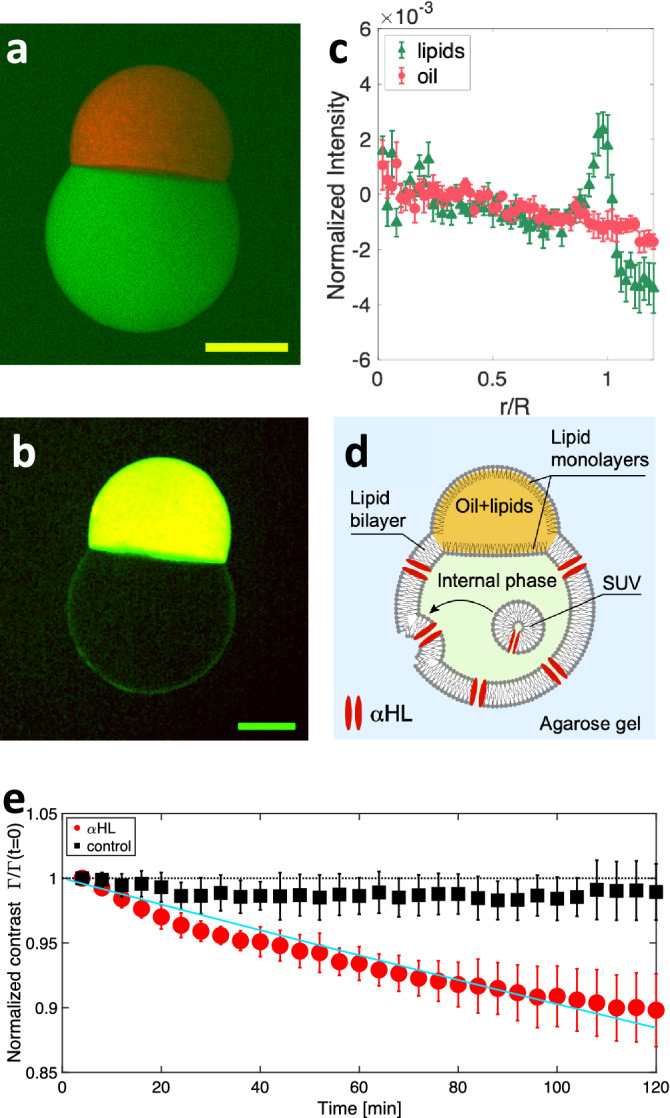
(**a**) Composite confocal microscopy image of a pseudo-vesicle trapped in an agarose gel. The internal phase contains carboxyfluorescein (green), while the oil phase contains Nile Red (red). (**b**) Composite image obtained by adding 1wt% fluorescent NBD-PC in the lipid mixture (green) and an oil containing Nile Red (red). Yellow color = red + green channels. For clarity, the image has been smoothed with a 2 pixel radius Gaussian filter. Scale bars = 200 $\mu$m. (**c**) Normalized radial fluorescence intensity profile, averaged over N=8 pseudo-vesicles labelled as in (**b**). The green triangles (*resp.* red disks) show the fluorescent lipids channel (*resp.* fluorescent oil channel). Error bars are SE of data. $R=316\pm 24$ $\mu$m is the average pseudo-vesicle size. (**d**) Sketch of $\alpha$HL insertion in the pseudo-vesicle membrane, using $\alpha$HL loaded SUV. (**e**) Time evolution of the normalized contrast $\Gamma (t)/\Gamma (t=0)$, for $\alpha$HL loaded pseudo-vesicles (red disks, N=5) and control experiments without $\alpha$HL (black squares, N=5). Error bars are SD of data. The solid line is an exponential fit of the data $\Gamma (t)/\Gamma (t=0) = e^{-t/\tau } \approx 1-t/\tau$, with $\tau = 5.9\pm 0.3~.10^4 s$.

To characterize the pseudo-vesicle and probe the existence of a lipid bilayer, we first used fluorescent markers dispersed in both the internal aqueous phase (carboxyfluorescein, emission wavelength $\lambda _c = 525$ nm, green, see “Methods” section) and the oil phase (Nile red, emission wavelength $\lambda _n = 636$ nm, red). The pseudo-vesicle is produced in the agarose gel as described above, and further imaged using a confocal microscope, with a 4x objective. The fluorescence images from both the green and red channels are recorded (see Fig. 2a), showing that the fluorescent water phase is efficiently encapsulated in the pseudo-vesicle. Secondly, we added green fluorescent lipids (NBD-PC, 1wt%) to the lipid mixture present in the oil phase labelled with Nile Red. A composite image obtained by confocal microscopy is shown on Fig. 2b. On Fig. 2c, we plot for each channel the normalized radial intensity profiles ($(I(r)-I(0))/(I_{\textrm{cap}}+I(0))$, where $I_{\textrm{cap}}$ is the intensity in the oil cap in a given channel. These profiles reveal a significant increase of the fluorescent signal arising from the lipids at the boundary between the inner aqueous phase and the outer agarose gel. On the contrary there is no detectable fluorescence signal arising from the oil phase, indicating that there is not a measurable layer of oil in the bilayer, at the confocal microscope spatial resolution and in the limit of our fluorescence imaging sensitivity. Altogether, our results confirm that the inner phase is efficiently encapsulated in a lipid bilayer devoid of significant amounts of oil. This doesn’t rule out the presence of oil traces at submicrometer length scales in the bilayer, as evidenced in emulsion template formed vesicles^33^. However, it was shown in^34^ that such oil residues in the membrane did not impair significantly the mechanical properties nor the functionality of the membrane. The membrane tension $\gamma _b$ can be estimated from the contact angles of the oil cap with the aqueous and agar phases (*see Supplementary Information*) and yields $\gamma _b = 4.15 \pm 0.33$ mN/m (obtained from 13 images on N=8 pseudo-vesicles), which is much larger than values usually reported in electroformed lipid vesicles^35,36^, $\gamma _b \sim 10^{-3}$ mN/m. On the contrary, our membrane tension value compares well with those obtained in Droplet Interface Bilayers (DIBs) which are planar lipid bilayers obtained by putting in contact two aqueous droplets bathing in an oil+lipid mixture (see for instance^37–39^). In DIBs, there is also water/oil interfaces surrounding the lipid bilayer. The high $\gamma _b$ value we measure in our pseudo-vesicle is thus, as in DIBs, presumably due to the presence of the oil cap which pins and stretches the bilayer on both sides.

### Nanopores insertion

To further probe the presence of a lipid bilayer and its functionality, we inserted $\alpha$-Hemolysin ($\alpha$HL) transmembrane proteins in the lipid bilayer. $\alpha$HL is an heptameric nanopore^40^ through which carboxyfluorescein molecules can diffuse^41^. Practically, we found that the direct dissolution of $\alpha$HL monomers in the internal aqueous phase was decreasing the pseudo-vesicle stability, due to a nanopore induced modification of the oil/water surface tension (See Supplementary Information). We therefore used Small Unilamellar Vesicles (SUV) containing $\alpha$HL nanopores, primarily prepared (see “Methods” section) and dispersed in the fluorescent aqueous internal phase loaded with carboxyfluorescein. The pseudo-vesicle is then formed as described above, allowing for the SUVs contained in the inner phase to fuse with the bilayer, as previously shown in^42^. This fusion allows in turn the insertion of functional transmembrane channels into the lipid bilayer^43,44^, $\alpha$HL in our case (Fig. 2d). Under an epifluorescence macroscope, we imaged the carboxyfluorescein leakage across the pseudo-vesicle by acquiring one image every 4 minutes, for two hours. We performed control experiments using on the one hand SUVs devoid of $\alpha$HL nanopores, and on the other hand pseudo-vesicles prepared without SUVs nor $\alpha$HL. Using image analysis, we measured the average intensity inside (*resp.* outside) the pseudo-vesicle $I_{in}$ (*resp.* $I_{out}$) from which we determine the fluorescence intensity contrast $\Gamma = (I_{in}-I_{out})/(I_{in}+I_{out})$. The time variation of the normalized contrast $\Gamma (t) / \Gamma (t=0)$ is presented on Fig. 2e. Clearly, the normalized contrast decreases over time for pseudo-vesicles containing $\alpha$HL whereas it remains constant for $\alpha$HL-free pseudo-vesicles. This establishes that the lipid bilayer can be functionalized with a transmembrane protein. More quantitatively, fitting the normalized contrast with an exponential decay (solid line on Fig. 2e), we obtain a characteristic release time of $5.9\pm 0.3~.10^4$ s. This timescale is expected to scale with the volume of the vesicle^45^. Taking into account our large vesicle size, our results are in good agreement with values reported in the literature^34,45,46^.

## Mechanical excitation

**Figure 3:**
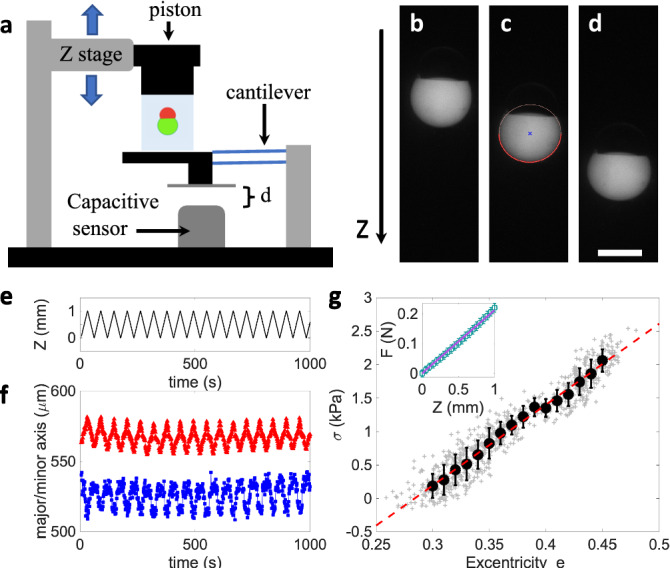
(**a**) Sketch of the mechanical excitation setup. (**b**-**d**) Macroscope fluorescence images of pseudo-vesicles either relaxed ((**b**) *Z*=0) or deformed (*Z*=0.5 and 1 mm in (**c**,**d**), respectively). In (**c**) the red line is the fit of the contour of the lower part of the pseudo-vesicle with an ellipse. Scale bar = 400 $\mu$m. (**e**) Piston position *Z*(*t*). (**f**) Major (red) and minor (blue) axes of the fitted ellipse as a function of time, for a 1000 s long cyclic indentation. (**g**) Compressive stress $\sigma$ as a function of the ellipse’s eccentricity *e*. Grey crosses correspond to all data points from (**f**), black circles are averages within bins of eccentricity $\delta e$= 0.01. Error bars are SD of the data. The red doted line is a linear fit to the data. Inset: Normal force as a function of the piston indentation. The line is a linear fit $F=\kappa z$, from which the gel compression modulus can be deduced, $K_{gel}=\kappa H/S$, with *H* the height of the gel.

Since the pseudo-vesicle is trapped within the bulk of the agarose gel, it can be deformed by indenting the surface of the gel (Fig. 3a). We compress the surface of the gel with a square piston (surface *S*=1 $\hbox {cm}^2$) mounted of a motorized *z*-translation stage (indentation amplitude, $\Delta$z = 1 mm, Fig. 3b–e), while measuring the applied normal force *F* (“Methods” section). With our fluorescence macroscope, we image a pseudo-vesicle containing carboxyfluorescein, as the pseudo-vesicle is cyclically deformed (Fig. 3b–d). Using image analysis, we fit an ellipse to the pseudo-vesicle shape (excluding the oil cap from the analysis, Fig. 3c). The time variation of the long *a* and short *b* axis of the ellipse are plotted on Fig. 3f, from which we compute the ellipse’s eccentricity $e = \sqrt{1-b^2/a^2}$. The variation of eccentricity, $\Delta e = e(F\ne 0)-e(F=0)$ can be used as a proxy for strain. On Fig. 3g, we plot the compressive stress $\sigma = F/S$ as a function of *e*. A linear relationship is observed, $\sigma = K_e \Delta e$, with an effective compression modulus of the pseudo-vesicle $K_e = 12.1\pm 0.6$ kPa. The value of $K_e$ is well distinct from the gel’s compression modulus $K_{gel} \approx 85$ kPa (see inset of Fig. 3g). In addition, we performed complementary experiments (*see Supplementary Information*) showing that the presence of the oil cap was not affecting the pseudo-vesicle deformation. Our method thus offer a easy-to-use platform to study lipid bilayer mechanics or mechanotransduction processes.

## Conclusion

Overall our approach constitutes a very simple method to produce and trap pseudo-vesicles within a gel, which is easy to set up and inexpensive. On the one hand, the method only requires small volumes of encapsulated phase ($\sim$ 1 $\mu$L of sample), in contrast with usual microfluidic techniques^21^ which usually require hundreds of microliters of solutions in order to obtain stable flows. On the other hand, it is based on gentle manipulation, thus avoiding any protein denaturation which can be caused by the application of electric fields^18,19^ as in electroformation techniques. It makes it of special interest for the use of valuable and delicate biological samples. Note, however, that the estimated value of the membrane tension in our system is much larger than values usually reported on electroformed vesicles, presumably due to the presence of the oil cap which pins and stretches the bilayer on both sides. This effect should be taken into consideration for further applications of the method. Last, because the pseudo-vesicles are trapped in a gel, they are easily localized and do not require post-production handling, in contrast with emulsion template methods. The pseudo-vesicles can also be easily deformed by indenting the gel surface, allowing to finely tune both the stress amplitude and frequency. Finally, the pseudo-vesicles are embedded in a viscoelastic gel which better mimics the mechanical properties of biological tissues.

## Methods section

### Chemicals

Unless specified, all chemicals were purchased from Sigma Aldrich, Merk inc.

#### Internal/external aqueous phase

The internal aqueous phase of the double droplet contains 10 mM Tris buffer (pH=7.5), 400 mM sucrose, and 100 mg/mL Dextran ($\hbox {M}_w$=40.10$^3$ g/mol,). For fluorescent pseudo-vesicles, we added 20 $\mu$M of carboxyfluorescein to this buffer. The osmolarity of the internal phase, measured with a Löser TYP6 osmometer is about 600 mOsm.

The external phase is made of a 3 wt% low gelling temperature Agarose, dissolved in a buffer containing 10 mM Tris (pH=7.5) and 200 mM potassium chloride. Prior to agarose addition we adjust the osmolarity to 380 mosm. Agarose is then dissolved in the buffer on a hot plate and the resulting solution is maintained in a liquid form at 85$^\circ$C.

#### Oil phase containing the phospholipids

The oil phase is a 50/50 wt/wt mixture of Hexadecan and Silicon oil AR20 in which dried lipids are dissolved. The lipid mixture is the one described in^45^ to mimic the bacterial membrane composition and maximize its stability in droplet interface bilayer geometries. It consists of 1,2-dioleoyl-sn-glycero-3-phosphocholine (DOPC, 81.1 wt%), 1,2-diphytanoyl-sn-glycero-3-phosphocholine (DPhPC, 10.8 wt%), 1,2-dioleoyl-sn-glycero-3-phospho-(1’-rac-glycerol) (sodium salt) (DOPG, 5.4 wt%) and cholesterol (2.7 wt%). Note that such a high concentration of DOPC prevents the formation of lipid domains both in the bilayer^47,48^ and at the oil/water interface^49^. In practice, we prepare a solution of 7.4 mg of this lipid mixture dissolved in chloroform. The lipids are then dried under nitrogen and kept under inert atmosphere in a 2 mL vial at -20$^\circ$C for several weeks. Immediately before use, we add 2 mL of the oil phase to yield a total lipid concentration of 3.7 mg/mL, and place the vial in an ultrasonic bath for 30 minutes at 30 $^\circ$C. This oil/lipid mixture can then be used for a few days.

For confocal imaging, we labelled separately the oil phase and the phospholipids. The oil phase is labelled with Nile Red with the following procedure: a small amount of solid Nile Red (typically the tip of a spatula) is dissolved in 300 $\mu$L of acetone. 5 mL of silicone oil is then poured onto the acetone and stirred overnight at room temperature in order to transfer the Nile Red dye into the oil phase while evaporating all traces of solvent.

For the labelling of the lipid bilayer, fluorescent lipids were incorporated in the lipid mix following the above-mentioned procedure. We use 1-Myristoyl-2-[12-[(7-nitro-2-1,3-benzoxadiazol-4-yl)amino]dodecanoyl]-sn-Glycero-3-Phosphocholine or NBD-PC lipids that are excited at 480 nm, allowing us to image simultaneously the oil phase labelled with Nile Red and the lipid bilayer labelled with NBD-PC. In that case the final lipid concentrations in the mix yield: 80.3wt% DOPC, 10.7wt% DPhPC, 2.7wt% Cholesterol, 5.4wt% DOPG, 1wt% NBD-PC.

#### SUV preparation

The above-mentioned lipid mixture (total lipid mass 1.25 mg) is dried under nitrogen in a test tube and left to dry further in a vacuum chamber overnight. The next day, 1 mL of the internal aqueous phase is added to the lipid film and the solution is placed in a probe sonicator for 30 minutes using on/off cycles of 15/5 seconds, respectively.

#### Preparation of $\alpha$HL solutions and integration in SUV

$\alpha$HL monomers are prepared in the internal aqueous buffer at a concentration of 250 $\mu$g/mL. In order to obtain SUV decorated with $\alpha$HL nanopores, 30 $\mu$L of this $\alpha$HL solution was added to 120 $\mu$L of the SUV solution, yielding a final pore monomer concentration of 50 $\mu$g/mL. The resulting mix is incubated at room temperature for about an hour and used as the inner phase of the pseudo-vesicles.

### Vesicle formation and mechanical excitation

The oil/aqueous phase sandwich is made following the steps described in the main text. The warm agarose solution is poured into a spectrophotometer cuvette (dimensions 10x10x40 $\hbox {mm}^{3}$). Temperature of the agarose is measured with a temperature thermocouple (USB-TC01, National Instrument). When the temperature reaches about 38 °C, the double droplet is formed. The system is left to cool down to room temperature for about 15 minutes, so that the agarose gels around the pseudo-vesicle. Note that the method can in principle be used in liquid (agarose-free) environments, which would allow to use oil-cap removal techniques as described in^22–24^. In our first trials, however, the high sedimentation velocities of the produced droplets (that were large, and significantly denser than the outer phase) were destabilizing the pseudo-vesicles during their formation. Reducing the pseudo-vesicle size (using a smaller injection tip diameter), adjusting the density of the inner phase or increasing the external phase viscosity will lower the sedimentation speed of the produced double droplet and sounds like a promising route to obtain stable pseudo-vesicles in liquid environments.

For mechanical excitation experiments in agarose, a thin rectangular sheet of Plexiglas (width 1 mm) is added on one side of the cuvette to cover its wall, prior to liquid agarose pouring. As the agarose is gelling, this sheet is carefully removed, leaving an empty space between the agarose gel and the cuvette wall. Indeed, due to Poisson effect, a lateral expansion of the gel occurs as it is vertically compressed. This empty space is required to allow for this lateral expansion and a proper elastic deformation of the gel.

The cuvette is placed and fixed on the device displayed in Fig. 3a. The piston has been 3D printed to fit the cuvette size and a thin Plexiglas sheet is glued to the piston face indenting the agarose surface. The piston is mounted on a translation stage controlled by a Newport LTA-HL linear actuator (*Z* resolution of 1 $\mu$m). As the piston indents the gel, it deflects the set of two planar cantilevers attached to the base of the sample holder. A capacitive sensor measures the deflection of the cantilevers. Knowing the stiffness of these cantilever (from previous calibration) we deduce the applied normal force *F* (the measurement noise on *F* is 1 mN).

The first step of the indentation experiments consists in determining the gel surface position. To this end, the *Z* position of the piston is lowered by steps of 0.1 mm, while measuring the normal force. As the force reaches 5 mN the ramp is stopped and this *Z* position is taken as the origin for Z coordinates.

Subsequently, we impose a cyclic deformation of the gels, by indenting the gel, from this surface position, by a value $\Delta Z$, by steps of 100 $\mu$m. At each step, the force is measured. The pseudo-vesicle is imaged at each indention step (using a pulse of blue light, duration 500 ms) with a Leica Macroscope and a Pointgrey camera (BFLY-U3-23S6C-C).

### Smaller pseudo-vesicle production

To reach smaller pseudo-vesicle sizes, we use a smaller injection tip. In practice we use a Polyurethan tubing (Phymep) with an outer diameter of 240 $\mu$m and an inner diameter of 130 $\mu$m. The tubing is connected to a 50 $\mu$L syringe (Hamilton), mounted on a home-made micro-injector. The pseudo-vesicles are produced in agarose gel with the fluorescent inner buffer and imaged through epifluorescence. We determine their sizes using image analysis and find an average diameter $D= 410 \pm 45 \mu$m (N=6). In principle, smaller sizes could be obtained by using a smaller injection tip. The only experimental difficulty will consist in forming the oil/water sandwich. The use of a commercial micro-injector will be required.

## Supplementary Information

Supplementary Information.

## Data Availability

All data generated or analyzed during this study are included in this published article and its supplementary information files. Correspondence and requests for raw data and materials should be addressed to E.W. or L.-L.P.

## References

[1] Wang X (2021). Versatile phospholipid assemblies for functional synthetic cells and artificial tissues. Adv. Mater..

[2] Jeong S, Nguyen HT, Kim CH, Ly MN, Shin K (2020). Toward artificial cells: novel advances in energy conversion and cellular motility. Adv. Funct. Mater..

[3] Noireaux V, Libchaber A (2004). A vesicle bioreactor as a step toward an artificial cell assembly. Proc. Natl. Acad. Sci..

[4] Niederholtmeyer H, Chaggan C, Devaraj NK (2018). Communication and quorum sensing in non-living mimics of eukaryotic cells. Nat. Commun..

[5] Pontani L-L (2009). Reconstitution of an actin cortex inside a liposome. Biophys. J ..

[6] Gavriljuk K (2021). A self-organized synthetic morphogenic liposome responds with shape changes to local light cues. Nat. Commun..

[7] Chan Y-HM, van Lengerich B, Boxer SG (2008). Lipid-anchored DNA mediates vesicle fusion as observed by lipid and content mixing. Biointerphases.

[8] Chan Y-HM, van Lengerich B, Boxer SG (2009). Effects of linker sequences on vesicle fusion mediated by lipid-anchored DNA oligonucleotides. Proc. Natl. Acad. Sci..

[9] Heuvingh J, Pincet F, Cribier S (2004). Hemifusion and fusion of giant vesicles induced by reduction of inter-membrane distance. Eur. Phys. J. E.

[10] Garamella J (2019). An Adaptive Synthetic Cell Based on Mechanosensing, Biosensing, and Inducible Gene Circuits. ACS Synth. Biol..

[11] Hindley JW (2019). Building a synthetic mechanosensitive signaling pathway in compartmentalized artificial cells. PNAS.

[12] Bangham AD, Horne R (1964). Negative staining of phospholipids and their structural modification by surface-active agents as observed in the electron microscope. J. Mol. Biol..

[13] Stein H, Spindler S, Bonakdar N, Wang C, Sandoghdar V (2017). Production of isolated giant unilamellar vesicles under high salt concentrations. Front. Physiol..

[14] Dominak LM, Keating CD (2007). Polymer encapsulation within giant lipid vesicles. Langmuir.

[15] Dominak LM, Keating CD (2008). Macromolecular crowding improves polymer encapsulation within giant lipid vesicles. Langmuir.

[16] Blanken D, van Nies P, Danelon C (2019). Quantitative imaging of gene-expressing liposomes reveals rare favorable phenotypes. Phys. Biol..

[17] Angelova MI, Dimitrov DS (1986). Liposome electroformation. Faraday Discuss. Chem. Soc..

[18] Chen W (2006). Electroconformational denaturation of membrane proteins. Ann. N. Y. Acad. Sci..

[19] Freedman KJ, Haq SR, Edel JB, Jemth P, Kim MJ (2013). Single molecule unfolding and stretching of protein domains inside a solid-state nanopore by electric field. Sci. Rep..

[20] Pautot S, Frisken BJ, Weitz D (2003). Production of unilamellar vesicles using an inverted emulsion. Langmuir.

[21] Li W, Zhang L, Ge X, Xu B, Zhang W, Qu L, Choi C-H, Xu J, Zhang A, Lee H (2018). Microfluidic fabrication of microparticles for biomedical applications. Chem. Soc. Rev..

[22] Deshpande S, Dekker C (2018). On-chip microfluidic production of cell-sized liposomes. Nat. Protoc..

[23] Deshpande S, Caspi Y, Meijering AE, Dekker C (2016). Octanol-assisted liposome assembly on chip. Nat. Commun..

[24] Krafft D, López Castellanos S, Lira RB, Dimova R, Ivanov I, Sundmacher K (2019). Compartments for synthetic cells: osmotically assisted separation of oil from double emulsions in a microfluidic chip. ChemBioChem.

[25] Evans E, Needham D (1987). Physical properties of surfactant bilayer membranes: Thermal transitions, elasticity, rigidity, cohesion and colloidal interactions. J. Phys. Chem..

[26] Kulin S (2003). Optical manipulation and fusion of liposomes as microreactors. Langmuir.

[27] Garten M (2017). Whole-GUV patch-clamping. PNAS.

[28] Robinson T (2019). Microfluidic handling and analysis of giant vesicles for use as artificial cells: A review. Adv. Biosyst..

[29] Deschamps J (2009). Dynamics of a vesicle in general flow. PNAS.

[30] Schäfer E (2015). Mechanical response of adherent giant liposomes to indentation with a conical AFM-tip. Soft Matter.

[31] Lin YC (2019). Force-induced conformational changes in Piezo1. Nature.

[32] Lira RB, Steinkühler J, Knorr RL, Dimova R, Riske KA (2016). Posing for a picture: Vesicle immobilization in agarose gel. Sci. Rep..

[33] Campillo C, Sens P, Köster D, Pontani L-L, Lévy D, Bassereau P, Nassoy P, Sykes C (2013). Unexpected membrane dynamics unveiled by membrane nanotube extrusion. Biophys. J ..

[34] Van de Cauter L, Fanalista F, Van Buren L, De Franceschi N, Godino E, Bouw S, Danelon C, Dekker C, Koenderink GH, Ganzinger KA (2021). Optimized cDICE for efficient reconstitution of biological systems in giant unilamellar vesicles. ACS Synth. Biol..

[35] Hochmuth F, Shao J-Y, Dai J, Sheetz MP (1996). Deformation and flow of membrane into tethers extracted from neuronal growth cones. Biophys. J ..

[36] Rädler JO, Feder TJ, Strey HH, Sackmann E (1995). Fluctuation analysis of tension-controlled undulation forces between giant vesicles and solid substrates. Phys. Rev. E.

[37] Stephenson EB, Korner JL, Elvira KS (2022). Challenges and opportunities in achieving the full potential of droplet interface bilayers. Nat. Chem..

[38] Taylor GJ, Venkatesan GA, Collier CP, Sarles SA (2015). Direct in situ measurement of specific capacitance, monolayer tension, and bilayer tension in a droplet interface bilayer. Soft Matter.

[39] Huang Y, Chandran Suja V, Amirthalingam L, Fuller GG (2022). Influence of salt on the formation and separation of droplet interface bilayers. Phys. Fluids.

[40] Gouaux JE (1994). Subunit stoichiometry of staphylococcal alpha-hemolysin in crystals and on membranes: A heptameric transmembrane pore. PNAS.

[41] Valet M (2019). Diffusion through nanopores in connected lipid bilayer networks. PRL.

[42] Lira RB, Robinson T, Dimova R, Riske KA (2019). Highly efficient protein-free membrane fusion: A giant vesicle study. Biophys. J ..

[43] Barriga HM, Booth P, Haylock S, Bazin R, Templer RH, Ces O (2014). Droplet interface bilayer reconstitution and activity measurement of the mechanosensitive channel of large conductance from Escherichia coli. J. R. Soc. Interface.

[44] Garten, M., Aimon, S., Bassereau, P. & Toombes, G. E. Reconstitution of a transmembrane protein, the voltage-gated ion channel, KvAP, into giant unilamellar vesicles for microscopy and patch clamp studies. *JoVE (J. Vis. Exp.)*, e52281 (2015). 10.3791/52281 PMC4354550 25650630

[45] Dupin A, Simmel FC (2019). Signalling and differentiation in emulsion-based multi-compartmentalized in vitro gene circuits. Nat. Chem..

[46] Lu L, Schertzer JW, Chiarot PR (2015). Continuous microfluidic fabrication of synthetic asymmetric vesicles. Lab Chip.

[47] Veatch SL, Keller SL (2003). Separation of liquid phases in giant vesicles of ternary mixtures of phospholipids and cholesterol. Biophys. J ..

[48] Sorre B, Callan-Jones A, Manneville J-B, Nassoy P, Joanny J-F, Prost J, Goud B, Bassereau P (2009). Curvature-driven lipid sorting needs proximity to a demixing point and is aided by proteins. Proc. Natl. Acad. Sci..

[49] Pontani L-L, Haase MF, Raczkowska I, Brujic J (2013). Immiscible lipids control the morphology of patchy emulsions. Soft Matter.

