# Supplementary material for: A simple method to make, trap and deform a vesicle in a gel
Source: Sci Rep. 2023 Apr 2;13:5375. doi: 10.1038/s41598-023-31996-9 (PMC10068607; doi:10.1038/s41598-023-31996-9)
Supplement: Supplementary file 1 — Supplementary Information. [file 41598_2023_31996_MOESM1_ESM.pdf]

A simple method to make, trap and deform a vesicle in a gel

Pierre Tapie,[1] Alexis M. Prevost,[1] Lorraine Montel,[1] Léa-Laetitia Pontani,[1,*] and Elie Wandersman[1,†]

[1]*Sorbonne Université, CNRS, Institut de Biologie Paris-Seine (IBPS), Laboratoire Jean Perrin (LJP), 4 place Jussieu, F-75005 Paris, France.*

SUPPLEMENTARY INFORMATION

Fluorescence imaging, image analysis

For the experiments with αHL nanopores, the pseudo-vesicles contain carboxyfluorescein and are imaged through epifluorescence with a Leica macroscope. Images (Fig. a) are acquired every 4 minutes and analyzed as follows. First the image is binarized and we determine the centroid (x_c, y_c) of the white region using the *regionprops* function of Matlab (Fig. b). The edges of the white region are determined and we fit a circle to the lower part $(y < y_c)$ of the pseudo-vesicle (see Fig. c). The internal (*resp.* external) intensity I_{in} (*resp.* I_{out}) is taken as the average $\langle I(r < 0.5R)\rangle$ (*resp.* $\langle I(r > R)\rangle$). The contrast is then computed as $\Gamma = \frac{I_{in}-I_{out}}{I_{in}+I_{out}}$. We plot on Fig. d the normalized contrast $\Gamma/\Gamma(t = 0)$ as a function of time, for all performed experiments, with or without αHL.

For the experiment with fluorescent oil (red channel) and fluorescent lipids (green channel), the pseudo-vesicles are imaged through confocal microscopy (Spinning Disc Xlight V2, Gataca systems) with a 4x objective. To determine the radial intensity profile, we first manually click on the green channel image to specify three points on the pseudo-vesicle membrane. The position of these three points is then automatically refined by looking at an intensity maximum in its vicinity (blue points in Fig. e). We determine the circle of radius R passing through these 3 points (red dotted line in Fig. e). The radial intensity is determined by averaging in a 3 pixel width ring, restricted to the bottom half of the pseudo-vesicle Fig. f. The intensity is normalized as $I_{norm} = \frac{I(r)-<I(r<0.5R)>}{I_{cap}+<I(r<0.5R)>}$, where $< I(r < 0.5R) >$ is a measure of the averaged intensity inside the pseudo-vesicle, and I_{cap} is the intensity in the oily cap region. Corresponding radial profiles for both red and green channels are plotted on Fig. g. Taking the average over 8 replicates gives the Fig. 2c of the main text.

Surface tension measurements

Oil-water surface tension

We have measured the surface tension between the internal phase and the oil+lipid phase using pending drop experiments. We used a Nemesys syringe pump to slowly grow a droplet of the internal aqueous phase through a thin cylindrical tip, inside the oil+lipid phase (*See* Fig. a). The droplet is imaged in transmission using a LED array and a binocular. Recorded images are analyzed (*See* Fig. b) using the *Drop Analysis* ImageJ Plugin [1]. Experiments have been performed in the absence or in the presence of αHL nanopores, up to a concentration of 100 μg/mL. We plot on Fig. c the variations of the measured surface tension γ_{ow} as a function of the nanopore concentration [αHL]. The value of γ_{ow} decreases tenfold, from ≈3 mN/m down to ≈0.3 mN/m as the nanopore concentration is increased, from 0 to 10 $\mu g/mL$. Above this concentration, γ_{ow} remains constant. This decrease of surface tension is due to the non-specific adsorption αHL monomers at the oil/water interface (note that this is different from the heptamerization of αHL monomers in lipid bilayers leading to the formation of a transmembrane pore). In our system, a consequence of this surface tension decrease is that the oil cap tends to wet and spread on the aqueous inner droplet (*See* Inset of Fig. b) . Experimentally, this wetting correlates with a strong decrease of the pseudo-vesicle stability. The success rate of stable pseudo-vesicle formation for [αHL]>20 μg/mL is below 1%, which hinders the use of this protocol to insert nanopores in the vesicle membrane. As an alternative, we used SUVs decorated with αHL nanopores, dispersed in the internal aqueous phase. In this case, αHL monomers are already heptamerized in the SUV membrane, which prevents them from decreasing too strongly the oil/water surface tension.

Membrane tension estimate

To estimate the tension in the membrane of the pseudo-vesicles, we write the force balance at the triple contact point :

$$\gamma_b = \gamma_{ao}\cos(\theta_{ao}) + \gamma_{wo}\cos(\theta_{wo}) \quad (1)$$

where γ_b, γ_{ao} and γ_{wo} are the bilayer, agarose/oil and aqueous/oil surface tensions, respectively, and θ_{ao}, θ_{wo} the corresponding contact angles, as defined on the sketch of Fig. S2 above. Measuring the contact angles from the confocal microscope image shown on Fig. S2d, and taking $\gamma_{wo} = \gamma_{ao}$=3.1 mN/m (see Fig. S2c, in absence of pores) we obtain $\gamma_b \approx 4.15 \pm 0.33$ mN/m (obtained from

13 images on N=8 pseudo-vesicles). This value can be compared to those obtained in Droplet Interface Bilayers (DIBs) wich are planar lipid bilayers obtained by putting in contact two aqueous droplets bathing in an oil+lipid mixture (see for instance [2]). In DIBs, there is also an oil reservoir in contact with the lipid bilayer. Our value of γ_b lies in the range of reported measured values for DIBs [3, 4]).

Mechanical deformation of the pseudo-vesicle

One could naturally wonder whether the presence of the oil cap modifies or not the mechanical response of the membrane part of the pseudo-vesicle. In DIBs, the lipid membrane is also connected to a quasi-infinite lipid reservoir surrounding the droplets in contact. As discussed by *Najem et al.* in [5] any mechanical tension applied to the membrane will relax, at long times, towards the equilibrium membrane tension γ_b, due to new lipids recruitment. This effect will become prominent at timescales longer than the typical lipid recruitment timescale, of the order of a few minutes [6]. At shorter timescales, however, this effect is negligible and additional tension can be exerted on the membrane, allowing to trigger the gating of mechanosensitive channels [5] .

To further prove that the oil cap is not affecting much the membrane mechanical response, we performed complementary experiments, comparing the pseudo-vesicle deformations to those of spherical cavities embedded in an elastic medium under similar mechanical forcing (see a sketch on Fig. S3 A). These cavities are obtained by injecting a drop of water (resp. glycerol) in a liquid Polydimethylsiloxane (PDMS)-filled cuvette. As the PDMS is cured at 65^oC for 2h, the water droplet evaporates yielding a gas cavity in the cured PDMS elastomer (Young's modulus $E \approx 2$ MPa). For a glycerol droplet on the contrary, we found that the liquid droplet remained after PDMS curing, yielding a liquid cavity. The system is then placed in the excitation setup shown in Fig. 3a and we image in bright field the deformation of the cavity at different applied normal forces F (Fig. S3a). Using image analysis, we deduce the radial displacement field $u_r(\theta)$ in polar coordinates,

$$u_r(\theta, F) = r(\theta, F) - r(\theta, F = 0) \quad (2)$$

where the polar angle θ is defined on Fig. S3b and $r(\theta, F = 0) \approx R$.

The deformation of a cavity in an infinite elastic medium under compression has been theoretically modelled by Eshelby in the 1950's [7, 8]. Following their work, one expects $u_r(\theta) \sim B \cos(2\theta)$, with B the amplitude of the radial displacement of the cavity which writes

$$B = \frac{\sigma R}{E} \quad (3)$$

where $\sigma = F/S$ is the compressive stress. The radial deformation fields u_r are plotted on Fig. S3c and d respectively for cavities and for the pseudo-vesicle. In the latter case, the analysis is restricted to the bottom part to exclude the oil cap. In both cases, the u_r curves are well fitted by the Eshelby model, yielding an amplitude of deformation B which increases linearly with the force (Fig. S3e). Rescaling the deformation amplitude B by the undeformed radius R and σ by the Young's modulus E, all curves fall on the same master curve, close to the identity $y = x$ curve (Fig. S3f). Interestingly, these experiments show that the deformation of the pseudo-vesicle can be described by that of a cavity in an elastic medium. Therefore, the effect of the oil cap does not seem prominent regarding the membrane deformation. In addition, the Eshelby cavity model gives a simple framework to predict the pseudo-vesicle deformation, if its radius or the elastic modulus of the surrounding medium are changed. At higher excitation frequencies, however, one could expect the visco-elasticity of the agarose gel to play a role, but we leave this aspect for future investigations.

---

* E-mail: lea-laetitia.pontani@sorbonne-universite.fr
† E-mail: elie.wandersman@sorbonne-universite.fr

[1] A. Daerr and A. Mogne, J. Open Res. Soft. **4** (2016).
[2] E. B. Stephenson, J. L. Korner, and K. S. Elvira, Nature Chemistry **14**, 862 (2022).
[3] G. J. Taylor, G. A. Venkatesan, C. P. Collier, and S. A. Sarles, Soft matter **11**, 7592 (2015).
[4] Y. Huang, V. Chandran Suja, L. Amirthalingam, and G. G. Fuller, Physics of Fluids **34**, 067107 (2022).
[5] J. S. Najem, M. D. Dunlap, I. D. Rowe, E. C. Freeman, J. W. Grant, S. Sukharev, and D. J. Leo, Scientific reports **5**, 1 (2015).
[6] S. Leptihn, O. K. Castell, B. Cronin, E.-H. Lee, L. Gross, D. P. Marshall, J. R. Thompson, M. Holden, and M. I. Wallace, Nature protocols **8**, 1048 (2013).
[7] J. D. Eshelby, Proceedings of the royal society of London. Series A. Mathematical and physical sciences **241**, 376 (1957).
[8] J. C. Jaeger, N. G. Cook, and R. Zimmerman, *Fundamentals of rock mechanics* (John Wiley & Sons, 2009).

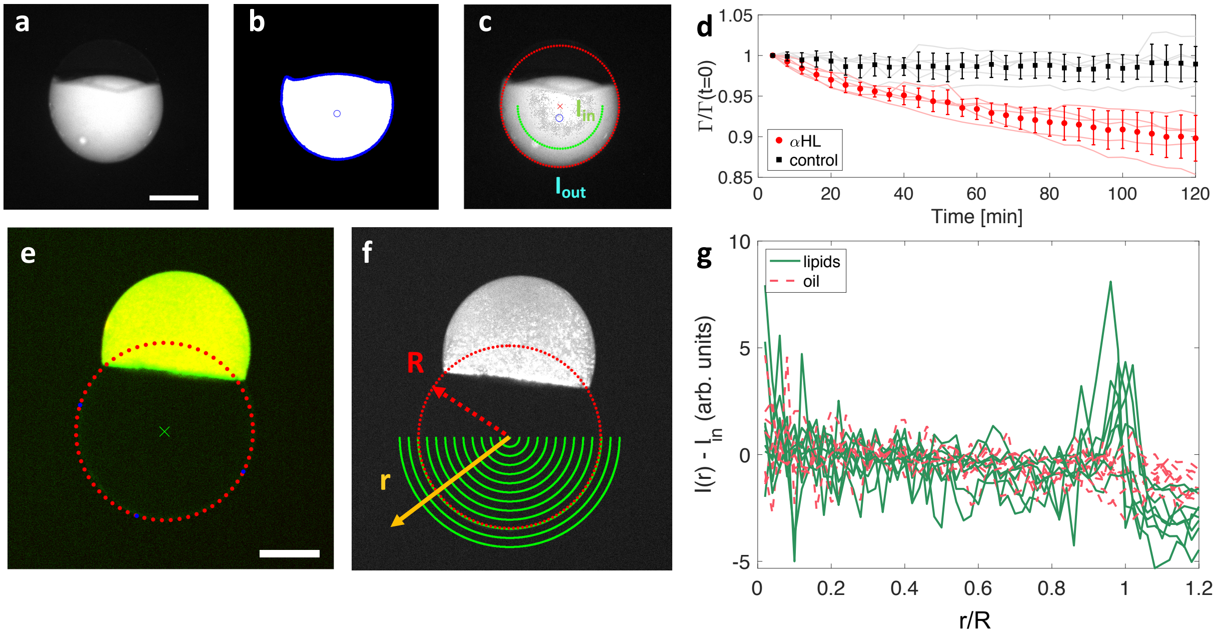

Figure S1 . a-c) Image analysis steps for contrast measurements. a) Raw epifluorescence imaging of a pseudo-vesicle. Scale bar = 200 μm. b) The image is binarized. The blue circle is the detected centroid of the white region. The edges of the binarized image are detected (blue dots). c) A circle is fitted to the lower part of the pseudo-vesicle's edge (red dots). The interior intensity is computed (within the green dashed semi-circle), the outside intensity is computed (within the blue half-rings). d) The normalized contrast is plotted over time. Solid lines represent individual experiments, symbols are averages (errorbars= standard deviation). e) Composite image of a pseudo-vesicle, with a fluorescent oil phase (Nile Red, red) and fluorescent lipids (NBD-PC, green). Yellow indicates the addition of the two channels. We manually click on three points of the pseudo-vesicle's membrane (blue disks) and determine the corresponding circle (red dotted line). f) Sketch of the image analysis procedure. Fluorescence intensities in both green and red channels are measured in radial half rings. g) Fluorescence intensity, to which the inner central intensity I_{in} has been subtracted, as a function of the radial coordinate, for both green and red channels.

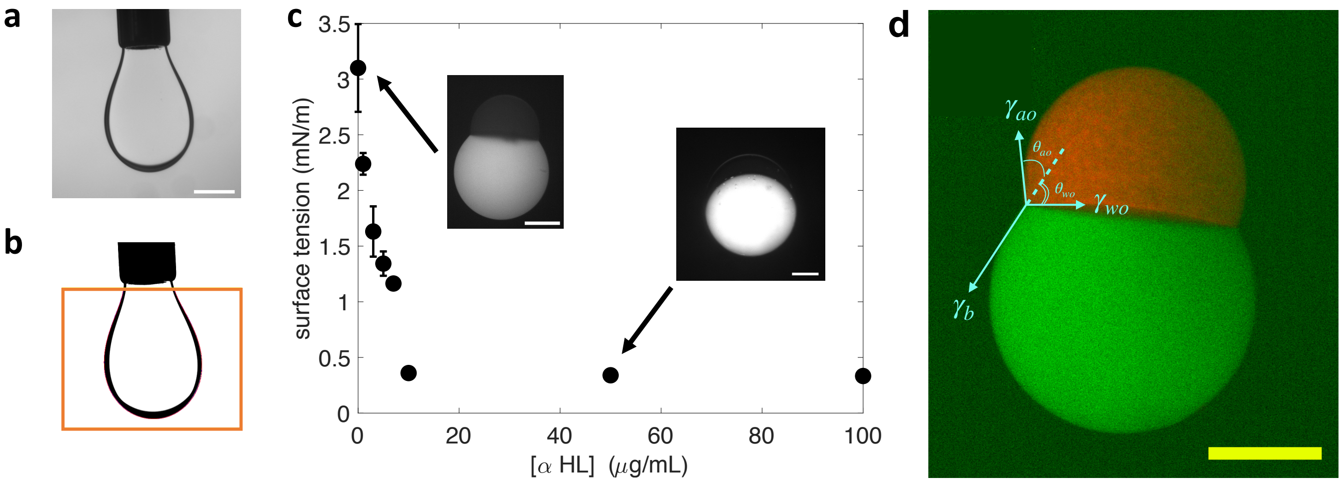

Figure S2 . a,b) Pending drop experiment with an aqueous droplet (internal phase, with [αHL] = 0.1 μg/mL) bathing in the oil+lipid mixture described above. In b) the image has been binarized and its shape (restricted to orange rectangle region of interest) is fitted to determine the surface tension, using the ImageJ plugin of [1]. Scale bar = 500 μm. c) Surface tension (deduced from the pending drops experiments) as a function of the nanopore concentration [αHL]. Inset: typical images of pseudo-vesicles trapped in an agarose gel, at [αHL]= 0 and [αHL]=50 μg/mL, as indicated by the arrows. d) Composite confocal microscope image of a pseudo-vesicle (oil marked in red, internal aqueous phase in green) on top of which the contact angles $\theta_{ao} \approx 39^o$ and $\theta_{wo} \approx 56^o$ have been drawn. Using Eq.1, the membrane tension γ_b can be estimated. The scale bar is 200 μm long.

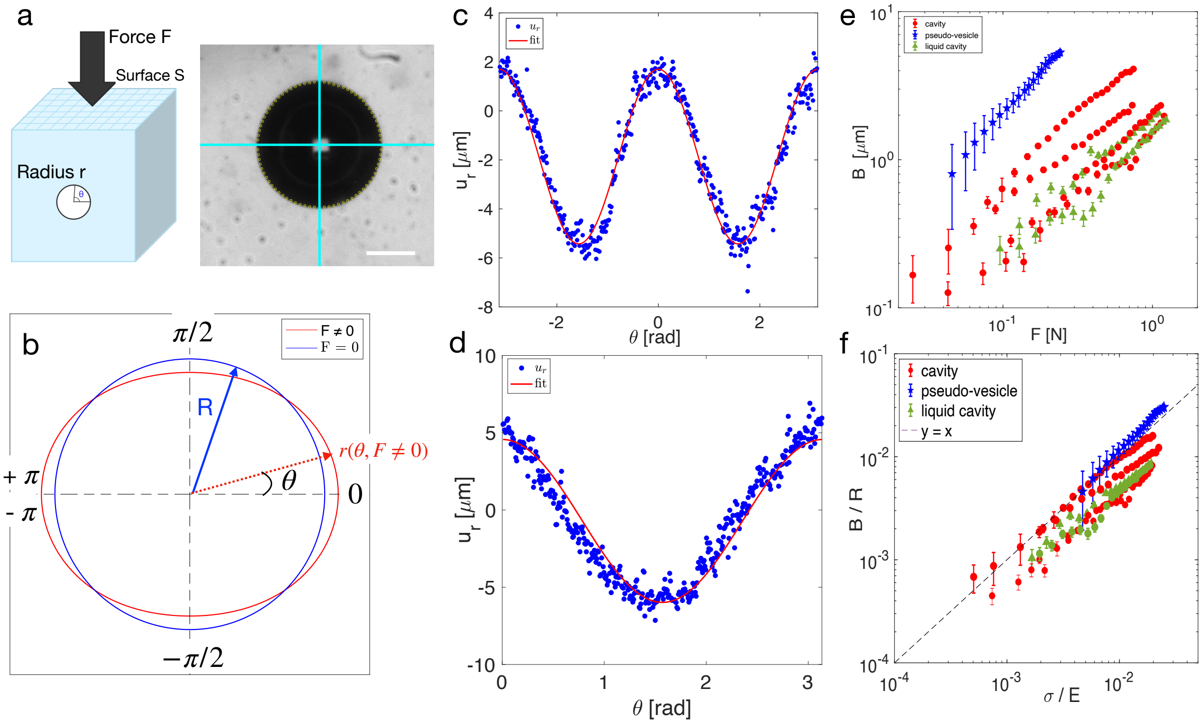

Figure S3 . a) Sketch of a cavity embedded in an elastic medium, upon uniform compression. Right image of the undeformed air cavity in a PDMS elastomer. The scale bar is 200 μm long. b) Sketch of a cavity under compression force. The radial coordinate $r(\theta, F \neq 0)$ is shown with a dashed red arrow. c) Radial displacement u_r as a function of the polar angle θ, for an air cavity in PDMS, under a force F=0.46 N. The red line is a fit $u_r = A + B\cos(2\theta)$. d) Same as c) for the pseudo-vesicle in an agarose gel, for F = 0.24 N. The analysis is here restricted to the bottom membrane part of the pseudo-vesicle. e) Maximum radial displacement B as a function of the applied force, for experiments with the air cavity (red disks), the pseudo-vesicle (blue pentagrams) and a liquid-filled cavity (green triangles). f) Rescaling of the previous graph, plotting B/R as a function of σ/E, where $\sigma = F/S$. The dashed line is the identity $y = x$
